# A Rare Case of I127V Heterozygous Transthyretin Amyloidosis With Atypical Transthoracic Echocardiogram Findings Presenting As Upper Extremity Sensorimotor Polyneuropathy

**DOI:** 10.7759/cureus.25259

**Published:** 2022-05-23

**Authors:** Maja Ostojic, Sukhraj S Gill, Jose David Avila, Brendan J Carry

**Affiliations:** 1 Internal Medicine, Geisinger Medical Center, Danville, USA; 2 Neurology, Geisinger Medical Center, Danville, USA; 3 Cardiology, Geisinger Medical Center, Danville, USA

**Keywords:** neurologic amyloidosis, cardiac amyloidosis, i127v, hattr, hereditary transthyretin, transthyretin amyloidosis

## Abstract

Hereditary transthyretin amyloidosis (hATTR) is a class of disorders with various systemic clinical manifestations, most often cardiac and neurologic in origin. The I127V mutation is a known but uncommon type of hATTR that typically affects males in their sixth or seventh decade of life. We present a case of this rare genetic variant with an atypical presentation of upper, followed by lower extremity sensorimotor polyneuropathy, with an uncharacteristic transthoracic echocardiogram (TTE) pattern but strongly positive pyrophosphate (PYP) scan, confirming the amyloidosis (AL) diagnosis.

## Introduction

Amyloidosis (AL) is a systemic disease of misfolded proteins with an incidence of 4,000 cases annually in the United States. The two broad classifications are light-chain amyloidosis and transthyretin amyloidosis (ATTR); the latter being further split into wild-type versus mutant [1]. The mutant ATTR most commonly deposits in the heart and/or nerves, and approximately 150 variants have been identified thus far [2]. TTR, formerly called prealbumin, is a clinically heterogeneous autosomal dominant protein gene [2] located on chromosome 18 and contains 127 amino acids. The most common mutation is the valine to methionine point mutation (Val30Met), accounting for about 50% of the TTR variants worldwide [1]. In turn, the valine to isoleucine point mutation (Val122Ile) is the most common in the United States, having a high association with cardiomyopathy and heart failure, mainly amongst African Americans [3]. However, in the literature thus far, there have been minimal (approximately 5) recorded findings of the valine for isoleucine substitution, Ile127Val, the unique mutation of our patient.

Only one amino acid substitution is needed to destabilize the TTR tetramer, promoting disaggregation of the protein and thus causing pathogenicity [3]. Normally, TTR serves as a transport protein for both thyroxine (T4) and retinol-binding protein. However, in this disease process, the extracellular deposition of amyloid fibrils leads to progressive multisystem dysfunction, mainly in the peripheral nerves and the heart [1, 4]. The neurologic symptoms initially present as numbness and neuropathic pain in the feet extending proximally. Pain and/or temperature sensation are usually affected before light touch and deep sensibility. Once the upper extremities are involved, a sensation mimicking bilateral carpal tunnel syndrome is very common. Autonomic impairment is also frequently seen [1, 5]. On the other hand, the cardiac symptoms usually present later in the disease course and demonstrate as symptoms of heart failure, mainly manifested due to loss of diastolic compliance and biventricular wall thickening [1].

Diagnosis may be difficult if there is no available family history to jumpstart the genetic screening process or raise clinical awareness of the possibility of the disease. This is because AL is rare and often presents nonspecifically, with initial symptoms possibly being as vague as fatigue or decreased exercise tolerance. Genetic testing (specifically, Sanger sequencing) or biopsy with subsequent Congo red staining, showing the apple-green birefringence under polarized light, is the mainstay of diagnosis [1]. A pathognomonic finding of cardiac disease is global longitudinal strain reduction with apical sparing seen via transthoracic echocardiogram (TTE) [6, 7], not seen in our patient. A pyrophosphate (PYP) scan is the only FDA-approved radiotracer in the United States to diagnose cardiac AL [8], which our patient was strongly positive for.

## Case presentation

A 59-year-old male with no significant past medical history presented with progressive symmetric bilateral hand numbness and weakness. His symptoms were initially attributed to bilateral carpal tunnel syndrome and cervical radiculopathy, which were treated with carpal tunnel release and cervical spine surgery. Symptoms initially improved but later recurred and progressively worsened. Several months later, the patient developed bilateral symmetric lower extremity numbness and weakness. Electromyography (EMG) was done, which revealed mixed motor and sensory polyneuropathy. Luckily enough, the patient had undergone voluntary genetic screening years prior. Through current research efforts, we were able to identify his medical chart with a high probability of TTR AL. This demonstrated the autosomal dominant heterozygous c.379 A> G p.I127V mutation in the TTR gene. Considering the degree of motor involvement and this particular variant that often mimics chronic inflammatory demyelinating polyradiculoneuropathy (CIDP), cardiology was involved in high suspicion of hATTR.

**Figure 1:**
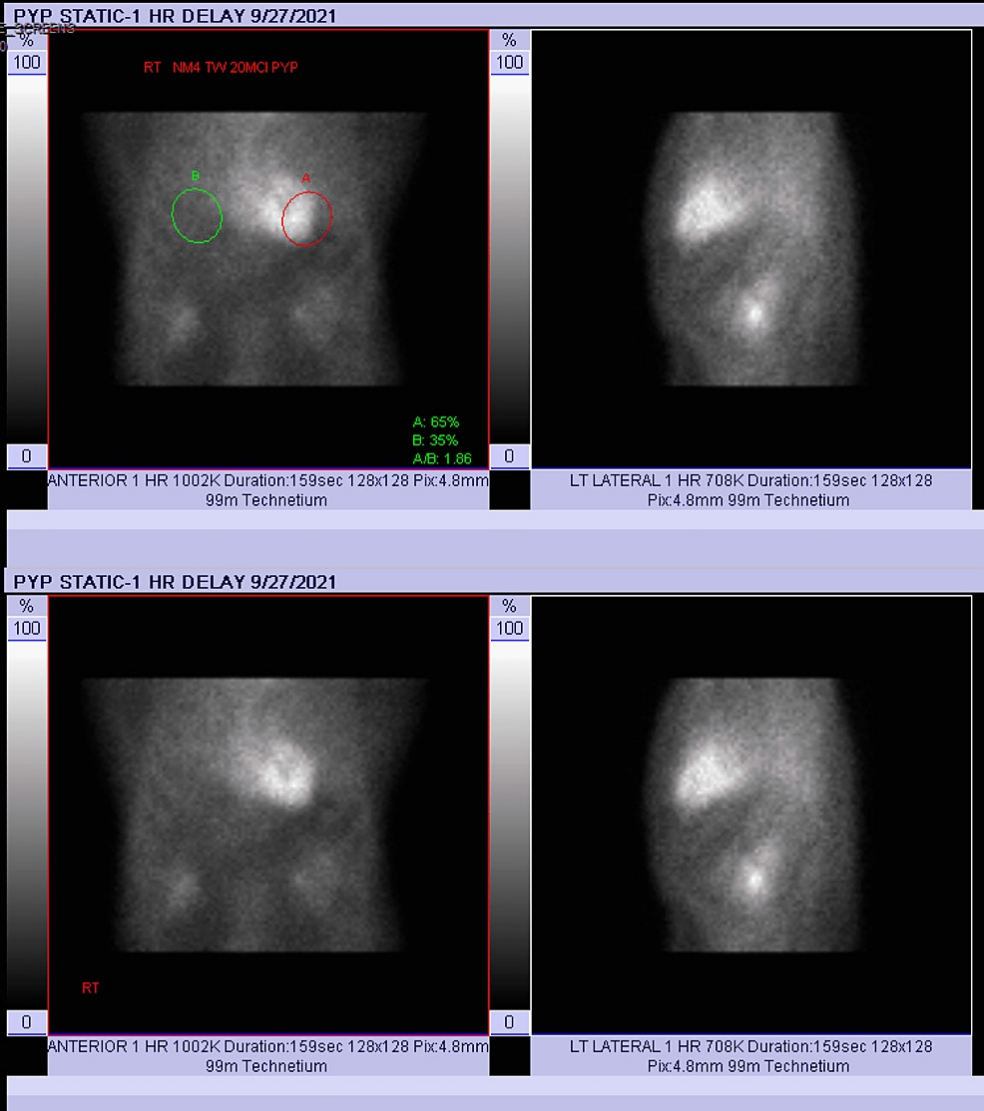
Positive pyrophosphate scan confirming amyloidosis.

TTE was performed, which showed a longitudinal strain pattern of 18%, suggesting normal left ventricular systolic function. However, the strain was uncharacteristically lower in the basal to mid inferior and inferoseptal regions. Interestingly, since the myocardium had no increased echogenicity, the TTE overall was not suggestive of amyloid. A PYP scintigraphy was completed and found to be Perugini Grade 3, suggesting strong positivity for cardiac AL. The patient was ultimately started on patisiran for polyneuropathy, with improvement in sensory symptoms, and later on tafamidis for cardiomyopathy (Figure 1).

## Discussion

Hereditary AL is a rare but severe autosomal dominant disorder of misfolded protein aggregation in mainly the cardiac and neurologic organ systems, with a prevalence of approximately 50,000 patients affected worldwide [4]. The I127V mutation has been even less prominent in literature, owing to the primary uniqueness of this case. The rarity of our case is also seen in the upper extremity involvement preceding lower extremity involvement months later. The classic presentation of PYP usually starts in the lower extremities and then spreads to the upper extremities [5]. Additionally, our patient presented with an uncharacteristic TTE pattern. Cardiac AL has the pathognomonic “cherry on top” (apical sparing) pattern, which was not seen in our patient [6]. This pattern is 93% sensitive and 82% specific for cardiac AL [7]. The confirmatory PYP scan was able to solidify the diagnosis, having a 91% sensitivity and 92% specificity for detecting ATTR cardiac AL [8]. Since symptom onset, this diagnosis is normally delayed by 4-5 years due to various misdiagnoses [2]. Therefore, early detection is imperative so that patients can be started on the appropriate therapy. Patisiran is a small interfering RNA molecule within a lipid nanoparticle that targets the TTR messenger mRNA, thereby degrading the misfolded protein [9]. Tafamidis, in turn, is a non-NSAID benzoxazole derivative that binds to TTR, slowing monomer formation and misfolding [10]. In various studies, both of these medications have been found to improve symptoms and halt disease progression [9, 10]. These two medications have had a significantly positive impact on our patient’s symptoms and disease burden.

## Conclusions

This is a rare case of I127V heterozygous TTR with an uncommon presentation of sensorimotor polyneuropathy with an uncharacteristic TTE pattern. The absence of the apical sparing pattern does not rule out cardiac AL, nor does the neuropathy pattern. AL is largely underdiagnosed overall. It is essential that genetic testing and the PYP scan always be considered in patients with suspected AL, even in the setting of atypical features. The available AL therapies have significantly improved patients’ quality of life and disease progression, making an early diagnosis of the disease crucial.
